# Balance between the two kinin receptors in the progression of experimental focal and segmental glomerulosclerosis in mice

**DOI:** 10.1242/dmm.014548

**Published:** 2014-04-17

**Authors:** Rafael Luiz Pereira, Raphael José Ferreira Felizardo, Marcos Antônio Cenedeze, Meire Ioshie Hiyane, Ênio José Bassi, Mariane Tami Amano, Clarice Sylvia Taemi Origassa, Reinaldo Correia Silva, Cristhiane Fávero Aguiar, Sylvia Mendes Carneiro, João Bosco Pesquero, Ronaldo Carvalho Araújo, Alexandre de Castro Keller, Renato C. Monteiro, Ivan Cruz Moura, Alvaro Pacheco-Silva, Niels Olsen Saraiva Câmara

**Affiliations:** 1 Laboratory of Clinical and Experimental Immunology, Nephrology Division, Federal University of São Paulo, São Paulo 04023-900, Brazil.; 2 Laboratory of Transplantation Immunobiology, Department of Immunology, Institute of Biomedical Sciences IV, University of São Paulo, São Paulo 05508-000, Brazil.; 3 Laboratory of Clinical and Experimental Immunology, Translational Medicine Division, Federal University of São Paulo, São Paulo 04039-002, Brazil.; 4 Laboratory of Cellular Biology, Instituto Butantan, Av. Vital Brazil 1500, São Paulo 05503-900, Brazil.; 5 Department of Biophysics, Federal University of São Paulo (UNIFESP), São Paulo 04023-062, Brazil.; 6 Department of Microbiology, Immunology and Parasitology, Federal University of São Paulo (UNIFESP), São Paulo 04023-062, Brazil.; 7 Institut National de la Santé et de la Recherche Médicale Unité Mixte de Recherche 699, Paris 75870, France.; 8 Instituto Israelita de Ensino e Pesquisa Albert Einstein, Renal Transplantation Unit, Albert Einstein Hospital, São Paulo 05521-000, Brazil.

**Keywords:** Focal and segmental glomerulosclerosis, Bradykinin receptors, Inflammation, Podocyte, Fibrosis

## Abstract

Focal and segmental glomerulosclerosis (FSGS) is one of the most important renal diseases related to end-stage renal failure. Bradykinin has been implicated in the pathogenesis of renal inflammation, whereas the role of its receptor 2 (B2RBK; also known as BDKRB2) in FSGS has not been studied. FSGS was induced in wild-type and B2RBK-knockout mice by a single intravenous injection of Adriamycin (ADM). In order to further modulate the kinin receptors, the animals were also treated with the B2RBK antagonist HOE-140 and the B1RBK antagonist DALBK. Here, we show that the blockage of B2RBK with HOE-140 protects mice from the development of FSGS, including podocyte foot process effacement and the re-establishment of slit-diaphragm-related proteins. However, B2RBK-knockout mice were not protected from FSGS. These opposite results were due to B1RBK expression. B1RBK was upregulated after the injection of ADM and this upregulation was exacerbated in B2RBK-knockout animals. Furthermore, treatment with HOE-140 downregulated the B1RBK receptor. The blockage of B1RBK in B2RBK-knockout animals promoted FSGS regression, with a less-inflammatory phenotype. These results indicate a deleterious role of both kinin receptors in an FSGS model and suggest a possible cross-talk between them in the progression of disease.

## INTRODUCTION

Kinins are powerful pro-inflammatory peptides (Bhoola et al., 1992; Campos and Calixto, 1995; Kang et al., 2004). Kinins are generated by the action of kallikrein enzymes on kininogen substrates. Two types of kallikrein enzymes are known, tissue and plasmatic kallikreins, which act on low-molecular-mass kininogens and high-molecular-mass kininogens, respectively (Regoli and Barabé, 1980; Campbell, 2001). The actions of these enzymes on their substrates generate bradykinin and kallidin, active peptides that signal through a constitutive G-protein-coupled receptor called the B2 receptor (B2RBK; also known as BDKRB2) (Bhoola et al., 1992; Marin Castaño et al., 1998). B2RBK is responsible for many of the physiological actions of kinins – such as decreasing blood pressure (Campos and Calixto, 1995), regulating blood flow, relaxing smooth muscle, enabling vascular permeability and nitric oxide release (Roman-Campos et al., 2010) and reducing oxidative stress (Xia et al., 2006). Despite its physiological role, B2RBK can also induce the activation of pro-inflammatory (Campos and Calixto, 1995; Marceau and Bachvarov, 1998; Calixto et al., 2000) and pro-fibrotic (Douillet et al., 2000; Stadnicki et al., 2005) cascades, which can, in turn, induce tissue damage.

Although B2RBK plays an important role in renal physiology per se, in some circumstances, the upregulation of B2RBK can lead to heterodimerization with the inducible B1 bradykinin receptor (B1RBK; also known as BDKRB1) (Barki-Harrington et al., 2003; Kang et al., 2004) and/or angiotensin type-1 receptor (AbdAlla et al., 2000; AbdAlla et al., 2001), contributing to the activation of these already known renal disease-related receptors (Wang et al., 2008; Wang et al., 2009; Pereira et al., 2011; Tunçdemir et al., 2011).

In the literature, we found different results concerning the role of B2RBK on fibrosis-related diseases, especially in renal tissue. Several papers describe a protective role for B2RBK (Wang et al., 2000a; Schanstra et al., 2002; Kakoki et al., 2010), whereas others describe a deleterious one (Dos Santos et al., 2008; Naito et al., 2010). Additionally, some groups report a dual role for B2RBK (Marin Castaño et al., 1998), which acts in physiological and inflammatory responses that are associated with cAMP and the release of phospholipase C, suggesting that there are different binding sites for the B2RBK ligand bradykinin, which could subsequently affect the signal transduction of this receptor. Our group has recently demonstrated the deleterious role of B1RBK in a focal and segmental glomerular sclerosis (FSGS) experimental model (Pereira et al., 2011). We also observed an upregulation of B2RBK. FSGS is one of the most important renal diseases that is related to end-stage renal failure; furthermore, an individual that is diagnosed with FSGS has a poor prognosis, which worsens when they present with high proteinuria. Most cases lead to end-stage renal disease within 5 years after the first signs of the illness (LeBrun et al., 2000; Seikaly et al., 2001; Franceschini et al., 2003; Deegens et al., 2008).

**TRANSLATIONAL IMPACT**

**Clinical issue**

Focal and segmental glomerulosclerosis (FSGS) is one of the major causes of end-stage renal diseases worldwide. FSGS is characterized by sclerotic lesions in glomeruli, and, at the clinical level, a classic hallmark is proteinuria (the presence of proteins in urine). Proteinuria is caused by an increase in permeability to proteins, which is induced by alterations in the structure and function of specialized glomerular cells called podocytes. Mutations in podocyte-related proteins, including nephrin and podocin, can give rise to FSGS; however, in most cases, the origin of disease is unknown. Experimental models of FSGS have been used since the 1980s and have helped to clarify many molecular aspects of the disease progression, such as the role of inflammation and the involvement of renin-angiotensin and kinin-kallikrein systems. Recent work showed the importance of kinin receptor 1 (B1RBK) in an experimental model of FSGS, providing incentive for further research into the role of kinin receptors in this disease.

**Results**

In this work, knockout animals and kinin receptor antagonists were used to unveil the role of kinin receptor 2 (B2RBK) in FSGS. The disease was induced in wild-type and B2RBK-knockout mice, using a previously established approach. In wild-type mice, blockage of the receptor with antagonists prevented FSGS when administered soon after disease induction and reversed signs of disease – including proteinurea – when administered during the later stages. Treatment with the B2RBK antagonist also downregulated fibrotic and inflammatory proteins that are associated with renal lesions. The authors report that FSGS is exacerbated in B2RBK-knockout mice, and, consistent with previous studies, higher B1RBK receptor expression was observed in these animals. Interestingly, treatment of B2RBK-knockout mice with a B1RBK antagonist ameliorated disease.

**Implications and future directions**

FSGS is associated with high morbidity and mortality worldwide, emphasizing the importance of searching for molecular targets that could reverse the clinical and histological features of, and even stabilize, disease progression. The results reported here indicate that kinin receptors are potentially important targets in FSGS, because their blockage with antagonists can restore podocyte architecture and protect against clinical symptoms, such as proteinuria. Although this work focused primarily on B2BRK, the data suggests cross-talk between the two receptors, which should be explored further in future studies. The understanding of molecular mechanisms provided by experimental models could help in the development of new therapeutic approaches against FSGS.

The role of B2RBK in the progression of FSGS is unknown, and few studies have reported it as a potential therapeutic target. Here, we unveil a role for this kinin receptor by demonstrating that B2RBK and B1RBK cross-talk in order to promote inflammation, resulting in an alteration of the permselectivity of glomerular membrane.

## RESULTS

### Early blockage of B2RBK protects animals from the first signs of FSGS

As detailed in recent papers (Pereira et al., 2011; Pereira et al., 2012; Reis et al., 2012), we have shown that the experimental model of FSGS is characterized by proteinuria, albuminuria, glomerulosclerosis and inflammation. Therefore, to evaluate the role of B2RBK in FSGS, we blocked this receptor by using the antagonist HOE-140 and then examined the effect on the aforementioned parameters. In the first protocol, we treated animals with HOE-140 at days 1, 2 and 3 after the injection of Adriamycin (ADM; also known as doxorubicin) and euthanized the animals at day 4, a timepoint at which proteinuria could be detected.

**Fig. 1.**
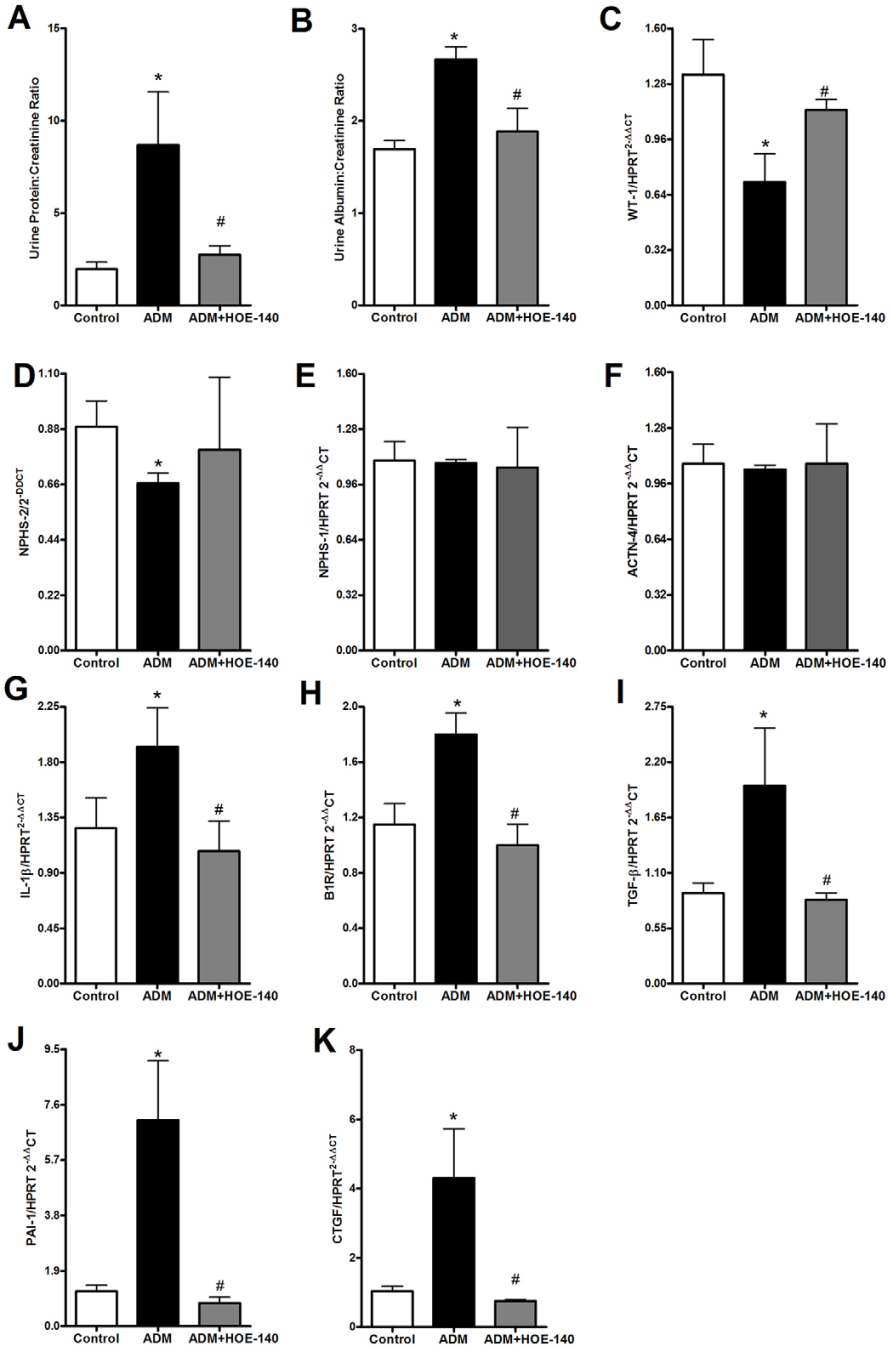
**Early HOE-140 treatment protects mice from the development of FSGS.** Four days after the injection of 10 mg of Adriamycin (ADM) per kg of bodyweight, Balb/c mice were killed. HOE-140 treatment protected mice from proteinuria (A) and albuminuria (B). HOE-140 also prevented the downregulation of the mRNAs encoding the podocyte proteins WT-1 (C) and podocin (NPHS-2) (D); however, HOE-140 did not affect the levels of nephrin (NPHS-1) (E) and α-actinin-4 (ACTN-4) (F) mRNA that were induced by ADM injection. The treatment also prevented the upregulation of mRNAs for the pro-inflammatory (IL-1β and B1RBK) (G,H) and pro-fibrotic markers TGF-β (I), PAI-1 (J) and CTGF (K). Expression of the mRNAs were normalized to that of hypoxanthine guanine phosphoribosyl transferase (HPRT). *P<0.05 compared with that of control mice, ^#^*P*<0.05 compared with that of mice treated with only ADM. Five animals were used per study group.

The early blockage of B2RBK protected animals from the proteinuria and albuminuria that was induced by the injection of ADM (Fig. 1A,B). The treatment was also effective at preventing the downregulation of the expression of the mRNAs encoding WT-1 (Fig. 1C) and podocin (also known as NPHS-2) (Fig. 1D); however, the treatment did not alter the expression of nephrin (also known as NPHS-1) or α-actinin-4 mRNAs (Fig. 1E,F).

The levels of pro-inflammatory cytokines have been linked to FSGS progression. In particular, the cytokine IL-1β has been shown to be one of the main molecules that is related to B1RBK expression (Klein et al., 2009; Pereira et al., 2011; Pereira et al., 2012; Reis et al., 2012). Therefore, we also quantified the expression of B1RBK mRNA. We observed that the treatment with HOE-140 was effective in downregulating the expression of IL-1β (Fig. 1G) and B1RBK (Fig. 1H) mRNAs, despite the fact that the renal levels of the IL-1β protein (supplementary material Fig. S1A) did not achieve statistical significance between the groups. The levels of other cytokines, such as tumor necrosis factor (TNF)-α and IL-17, showed no difference between the groups (supplementary material Fig. S1B–D).

Another important parameter to evaluate in FSGS is the renal expression of pro-fibrotic proteins. We observed that TGFβ-1 (hereafter referred to as TGF-β), plasminogen activator inhibitor type 1 (PAI-1; also known as SERPINE1) and connective tissue growth factor (CTGF) mRNA levels were downregulated after treatment with HOE-140 (Fig. 1I–K). The renal histology analysis at day 4 did not show any significant difference in the segmental sclerosis index, but the animals that had been treated with HOE-140 presented less mesangial hypercellularity, an important marker of FSGS progression (supplementary material Fig. S2A–D). Owing to the importance of macrophage infiltration in FSGS (Diamond and Pesek-Diamond, 1991; Ohtaka et al., 2002), we evaluated the renal protein levels of macrophage chemokines, but at this timepoint, the groups presented no significant difference between them (supplementary material Fig. S2E–H).

### Delayed blockage of B2RBK with HOE-140 reverses FSGS

**Fig. 2.**
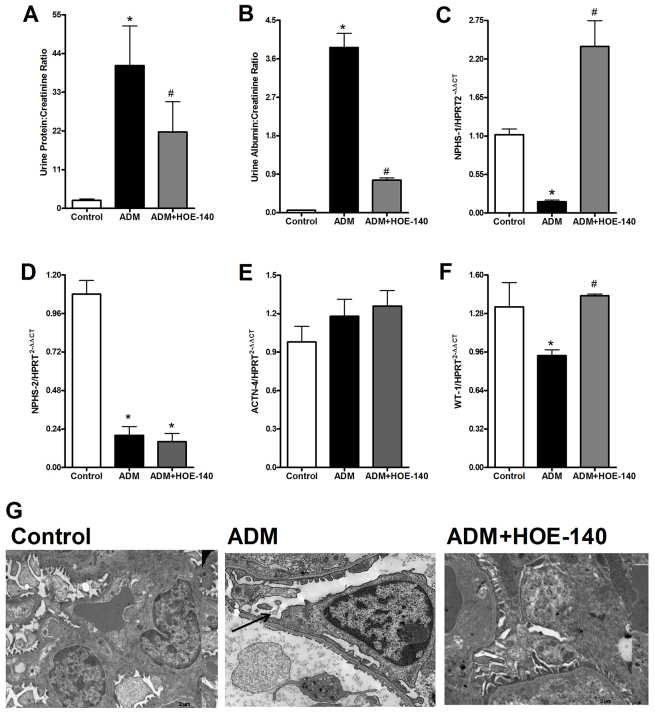
**Delayed HOE-140 treatment protects mice from the development of FSGS.** At day 7 after treatment with 10 mg of Adriamycin (ADM) per kg of bodyweight, balb/c mice were killed. Treatment with HOE-140 protected mice from proteinuria (A) and albuminuria (B). HOE-140 also prevented the downregulation of the mRNAs that encode the podocyte proteins nephrin (NPHS-1) (C), and WT-1 (F), although the treatment showed no difference in podocin (NPHS-2) (D) and α-actinin-4 (ACTN-4) (E) between groups. Expression of the mRNAs were normalized to that of hypoxanthine guanine phosphoribosyl transferase (HPRT). (G) We observed, by using electron microscopy analysis, that HOE-140 prevented the podocyte foot process effacement that was induced by ADM injection. The black arrow indicates podocyte effacement. *P<0.05 compared with that of control mice, ^#^*P*<0.05 compared with that of mice treated with only ADM. Five animals were used per study group.

In this protocol, we treated animals with HOE-140 at days 4, 5 and 6 after ADM injection. At this timepoint, signs of FSGS – such as albuminuria, downregulation of podocyte-related proteins and upregulation of inflammatory related cytokines (Pereira et al., 2011) – were already established. The animals were then euthanized at day 7. Treatment with HOE-140 diminished the ADM-induced proteinuria and albuminuria (Fig. 2A,B) that is associated with FSGS. We observed the protection of podocyte structure and the preservation of nephrin and WT-1 mRNA expression (Fig. 2C,F), but no differences were found in the expression of mRNAs encoding podocin and α-actinin-4 (Fig. 2D,E). Furthermore, animals that had been treated with HOE-140 showed less podocyte damage, as observed by using electron microscopy (Fig. 2G).

**Fig. 3.**
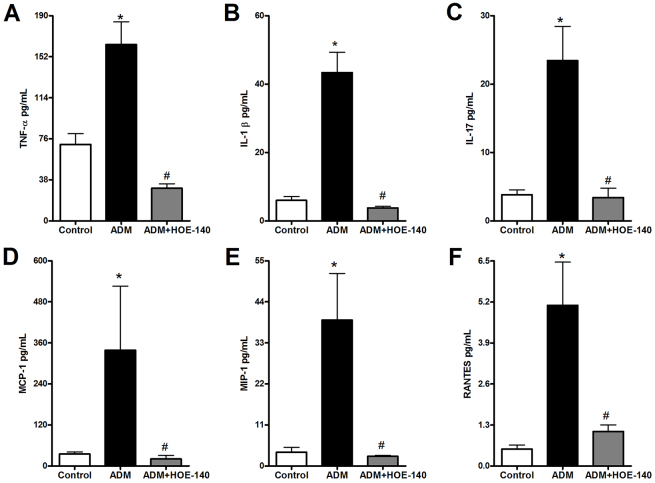
**Delayed treatment with HOE-140 protects mice from renal expression of pro-inflammatory and macrophage-related proteins that are induced by Adriamycin injection.** Parameters were analyzed at 7 days after the injection of Adriamycin (ADM). HOE-140 prevented the upregulation of the expression of TNF-α renal protein (A) and renal protein expression of IL-1β (B) and IL-17 (C). The blockage of B2RBK also efficiently prevented the upregulation of the renal expression of macrophage-related chemokines, such as MCP-1 (D), macrophage inflammatory protein 1 α (MIP-1; also known as CCL3) (E) and RANTES (regulated on activation, normal T cell expressed and secreted; also known as CCL5) (F). *P<0.05 compared with that of control mice, ^#^*P*<0.05 compared with that of mice treated with only ADM. Five animals were used per study group.

**Fig. 4.**
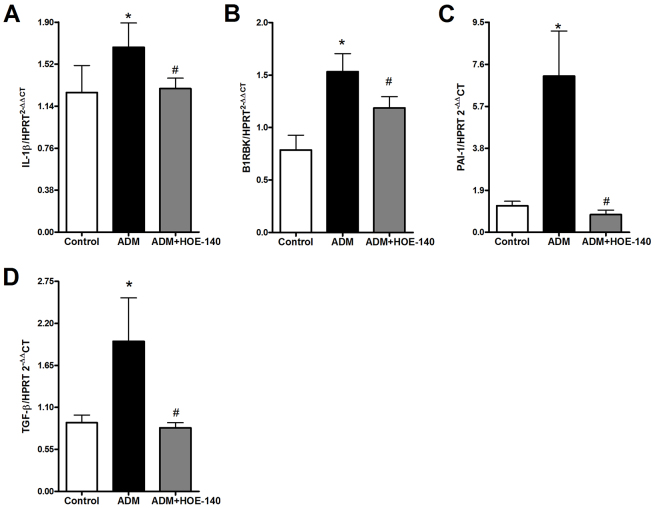
**Delayed HOE-140 treatment protects mice from renal expression of mRNAs that encode pro-inflammatory and pro-fibrotic molecules that are induced by Adriamycin injection.** The parameters were analyzed at 7 days after the injection of 10 mg of Adriamycin (ADM) per kg of bodyweight into balb/c mice. HOE-140 prevented the upregulated expression of IL-1β mRNA (A). The blockage of B2RBK was also efficient in preventing the upregulation of B1RBK mRNA expression (B) and the pro-fibrotic molecules PAI-1 (C) and TGF-β (D). Expression of the mRNAs were normalized to that of hypoxanthine guanine phosphoribosyl transferase (HPRT). *P<0.05 compared with that of control mice, ^#^*P*<0.05 compared with that of mice treated with only ADM. Five animals were used per study group.

Using HOE-140, we observed that blockage of B2RBK efficiently reversed the upregulation of the expression of pro-inflammatory cytokine proteins (Fig. 3A–C), including TNF-α, IL-1β and IL-17. The upregulation of IL-1 β mRNA expression was also abrogated upon B2RBK blockage (Fig. 4A). Interestingly, B2RBK blockage inhibited the expression of B1RBK mRNA (Fig. 4B).

Because macrophage infiltration is a common finding in FSGS experimental models (Diamond and Pesek-Diamond, 1991; Ohtaka et al., 2002; Pereira et al., 2011), we evaluated the role of B2RBK blockage on the renal expression of macrophage-related proteins (Fig. 3D–F) and also on the expression of monocyte chemoattractant protein 1 (MCP-1, also known as CCL2) mRNA (supplementary material Fig. S3A).

Fibrotic markers, such as PAI-1 and TGF-β, were also downregulated in mice that had been treated with HOE-140 (Fig. 4C,D).

Finally, the group that had been treated with HOE-140 showed less tubular damage and renal sclerosis, as observed by renal histology (supplementary material Fig. S3B–E).

### HOE-140 induces sustained protection during FSGS progression

After evaluating the efficacy of treatment with HOE-140 in the first two short-term protocols, we evaluated the same protocols over a longer term – after 21 days of ADM injection, a phase that is characterized by extensive sclerosis and renal damage.

**Fig. 5.**
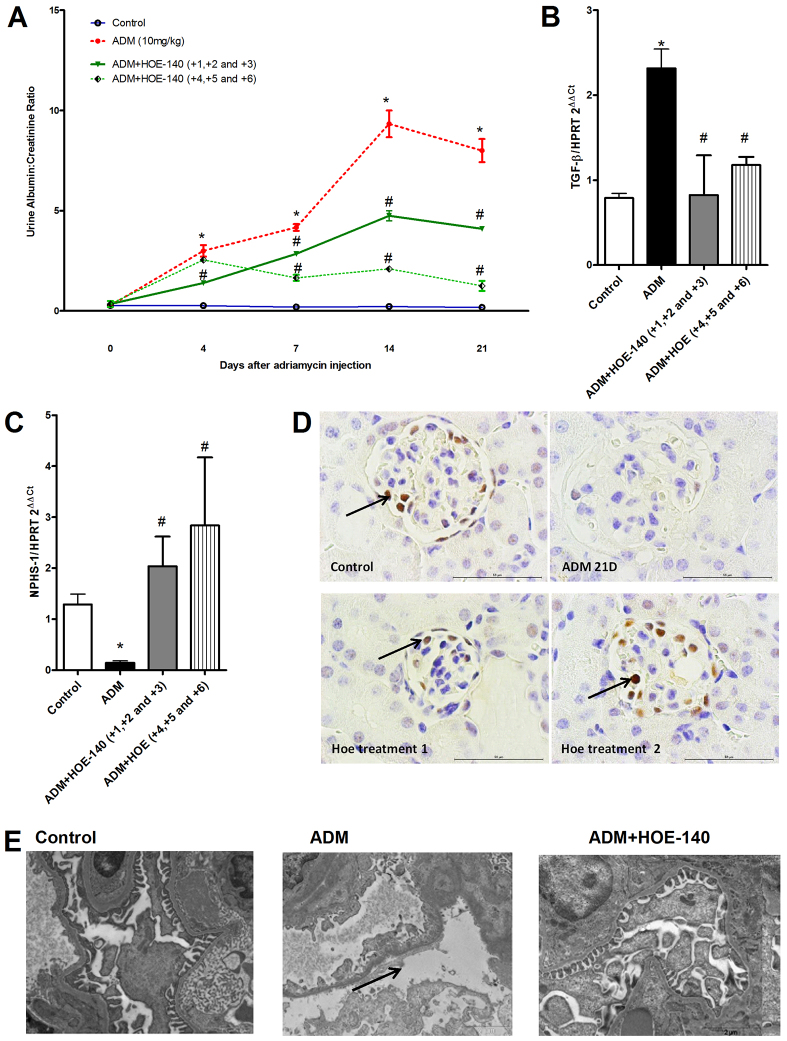
**Treatment with HOE-140 induced sustained protection of mice from FSGS development.** The balb/c mice were divided into two groups. In the first group, the mice were treated with HOE-140 at days 1, 2 and 3 after the injection of Adriamycin (ADM) (early treatment), and in the second group, the mice were treated with HOE-140 at days 4, 5 and 6 after the injection of ADM (delayed treatment). At the end of the two protocols, the mice were killed at day 21. Treatment with HOE-140, in both groups, protected mice from albuminuria (A). HOE-140 also prevented the upregulation of TGF-β mRNA (B) and prevented the downregulation of nephrin (NPHS-1) (C). Expression of the mRNAs were normalized to that of hypoxanthine guanine phosphoribosyl transferase (HPRT). HOE-140 also prevented the downregulation of the WT-1 staining that was induced by ADM injection, as observed by immunohistochemistry (D). ADM 21D, day 21 after injection of ADM; HOE treatment 1, early treatment; HOE treatment 2, delayed treatment. Black arrows indicate WT-1-positive cells. Scale bars: 50 μm. (E) By using electron microscopy analysis, we observed that HOE-140 prevented the podocyte foot process effacement that was induced by Adriamycin injection. Black arrow indicates podocyte effacement. *P<0.05 compared with that of control mice, ^#^*P*<0.05 compared with that of mice treated with only ADM. Five animals were used per study group.

Both protocols were efficient in preventing proteinuria (data not shown) and albuminuria (Fig. 5A). The expression of fibrotic markers, such as TGF-β mRNA, was also downregulated in HOE-140-treated animals (Fig. 5B). We observed that HOE-140 prevented the downregulation of podocyte proteins (Fig. 5C,D) and foot process effacement (Fig. 5E).

### Overexpression of B1RBK in B2RBK-knockout animals is associated with FSGS exacerbation

We further used genetically modified animals to study the role of B2RBK in FSGS. As many reports have shown (Wang et al., 2000b; Fogo, 2003; Pereira et al., 2011; Pereira et al., 2012; Reis et al., 2012), the experimental models of FSGS are predominantly generated in the Balb/c mouse strain. However, recent papers have demonstrated the possibility of adapting this method to establish the disease in Black/6 mice, thus raising new possibilities (Jeansson et al., 2009).

**Fig. 6.**
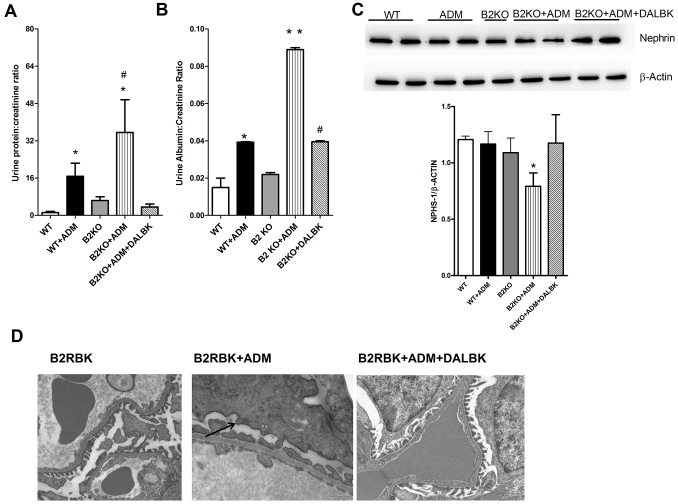
**The lack of B2RBK receptor exacerbates Adriamycin nephropathy.** C57-black-background mice were killed at day 7 after Adriamycin (ADM) injection. The lack of B2RBK (B2KO) exacerbates the ADM-induced proteinuria (A) and albuminuria (B), and these effects were downregulated by treatment with DALBK. ADM injection downregulated nephrin (NPHS-1) protein expression in B2RBK-knockout mice, and this downregulation was prevented by DALBK (C), as shown by western blotting (upper panel). The lower panel shows the quantification of the western blot analyses, where the level of nephrin was normalized to that of β-actin for each sample. (D) We observed that DALBK protects B2RBK-knockout mice from podocyte foot process effacement, by using electron microscopy. The black arrow indicates podocyte effacement. *P<0.05 compared with that of control mice, **P<0.05 vs all other groups, ^#^*P*<0.05 compared with that of mice treated with only ADM. Five animals were used per study group.

As Fig. 6A,B shows, B2RBK-knockout animals were more prone to developing ADM-induced FSGS because the animals showed increased proteinuria and albuminuria compared with that in wild-type mice in the same background. These results were supported by the downregulation of podocyte-related proteins and more podocyte foot process effacement and fusion in the knockout mice (Fig. 6C–E).

Previous data has shown that the B2RBK-knockout animals present with increased expression of B1RBK, which has devastating effects when activated in FSGS (Pereira et al., 2011). Initially, we confirmed that these animals highly expressed B1RBK (supplementary material Fig. S4A) and lacked B2RBK expression (supplementary material Fig. S4B). Finally, to examine the possible cross-talk between these two receptors, we blocked B1RBK with its specific antagonist DALBK in B2RBK-knockout animals. Surprisingly, we observed signs of protection from FSGS, including a reduction in podocyte foot process effacement and pro-fibrotic TGF-β expression (Fig. 6 and supplementary material Fig. S4).

## DISCUSSION

Recently, the deleterious role of B1RBK in FSGS has been described, where the activation of this receptor was associated with the upregulation of fibrotic and pro-inflammatory cytokines, such as TGF-β and IL-1β. IL-1β is one of the principal inducers of this receptor. Additionally, downregulation of podocyte proteins and podocyte foot process effacement was observed (Niemir et al., 1997; Kim et al., 2003; Schramek et al., 2009; Lee and Song, 2010; Lee, 2012). In this work, we focused our attention on the role of B2RBK, the other receptor that is associated with kinin signaling. In the literature, different results concerning renal kinin receptor blockage, or the lack of this receptor, have caused debate (Marin Castaño et al., 1998; Wang et al., 2000a; Schanstra et al., 2002; Dos Santos et al., 2008; Kakoki et al., 2010). In the first part of this work, we blocked B2RBK with the specific antagonist HOE-140 in the classic model of FSGS. Our treatments were performed according to the level of kinin receptor expression during FSGS (Pereira et al., 2011; Pereira et al., 2012). First, we blocked B2RBK at the beginning of disease progression. We observed, as some papers have shown (Braun et al., 2002; Dos Santos et al., 2008; Naito et al., 2010), that similar to B1RBK, B2RBK blockage can control proteinuria and albuminuria levels in animals. Furthermore, this positive result was reinforced by the maintenance of podocyte-related protein expression, which is, normally, downregulated in FSGS human and experimental models (Saleem et al., 2002; Pereira et al., 2011; Zheng et al., 2012). Associated with this protection, we observed the reduced expression of fibrosis-related proteins – such as TGF-β, PAI-1, vimentin and CTGF – which are usually upregulated in renal disorders (Chang et al., 2009; Klein et al., 2009; Ng et al., 2009). Another important finding of this treatment protocol was the downregulation of an important cytokine that is found in FSGS – IL-1β, which is linked to B1RBK upregulation (Ahluwalia and Perretti, 1996; Phagoo et al., 2001). These results demonstrate that HOE-140 could indirectly favor B1RBK downregulation, which can contribute to a better outcome of FSGS. Previous data have already indicated that B1RBK downregulation is associated with lower expression levels of TNF-α and IL-1β after treatment with HOE-140 (Abraham et al., 1991; Bandeira-Melo et al., 1999; Souza et al., 2003; Sainz et al., 2004; Souza et al., 2004). This possible cross-talk between the two receptors requires further investigation.

The second delayed-treatment protocol led to a better disease outcome, primarily because the animals had less proteinuria and albuminuria, and increased preservation of podocyte structure (as observed by electron microscopy), markers that are associated with better prognosis in FSGS (Wagner et al., 2008). Furthermore, the animals had less macrophage infiltration and renal and tubular lesions, other important biomarkers of FSGS progression (Klein et al., 2009; Pereira et al., 2011).

Finally, to evaluate the effects of long-term treatment with HOE-140, we treated the animals as in the first two protocols but then killed them at day 21, a timepoint at which the disease has significantly progressed, as observed in other papers that have been published by our group (Pereira et al., 2011; Pereira et al., 2012; Reis et al., 2012). Although all treatments reduced FSGS progression, the long-term treatment with HOE-140 in the final experiments led to reduced levels of sclerosis and albuminuria; we have observed previously that peaks in the levels of sclerosis and albuminuria correlated with the peaks of B2RBK expression in FSGS (Pereira et al., 2011). Therefore, higher levels of this receptor are associated with disease progression, suggesting cooperative signaling of kinin receptors in the development of FSGS. In particular, B2RBK blockage was associated with B1RBK downregulation, as observed previously (Seguin et al., 2008; Klein et al., 2009).

To understand the conflicting results in the literature when B2RBK-knockout animals are used, we evaluated markers of FSGS in these animals. Because B2RBK-knockout animals have been previously generated in the C57 black/6 background, we adapted our model into these animals, as performed previously by Jeansson and colleagues (Jeansson et al., 2009).

To our surprise, B2RBK knockout had different results compared with blockage of B2RBK. These animals showed increased albuminuria, increased podocyte foot process effacement, downregulation of podocyte-related proteins, upregulation of fibrotic molecules and increased glomerulosclerosis. Taken together, these results indicated that blockage of B2RBK is associated with protection from FSGS and that B2RBK-knockout animals are more prone to developing the disease.

The key difference between the two analyses seems to be the expression of B1RBK in these treatments. B1RBK has been extensively associated with kidney disease progression (Christopher and Jaffa, 2002; Klein et al., 2009; Wang et al., 2009), and inflammatory and fibrotic states (Ahluwalia and Perretti, 1996; Ricupero et al., 2000; Ni et al., 2003; Westermann et al., 2008; Westermann et al., 2009). Furthermore, polymorphisms in both receptors have been associated with renal diseases (Bachvarov et al., 1998; Maltais et al., 2002; Zychma et al., 2003). B2RBK blockage with HOE-140 seems to be associated with B1RBK downregulation; however, we observed B1RBK upregulation in B2RBK-knockout animals, suggesting that B1RBK expression compensates for the loss of B2RBK, as has been observed previously by other groups (Xia et al., 2006; Klein et al., 2009; Kakoki et al., 2010). To test our hypothesis, we blocked B1RBK in B2RBK-knockout animals. To our surprise, the blockage of B1RBK attenuated FSGS progression, and the animals showed less podocyte foot process effacement.

Although there is little data in the literature concerning the role of kinin receptors in FSGS, our work provides important information that contributes to a better understanding of the complex role of kinin receptors in FSGS, an exciting area in which we hope to instigate further studies. This work primarily focused on the role of B2RBK; however, we conclude that when the mRNAs for both receptors are upregulated, both of the kinin receptors can contribute to disease progression. Cross-talk between the receptors might be mediated by the upregulation of pro-inflammatory and pro-fibrotic molecules, which are closely related to FSGS progression. Finally, care should be taken when kinin-receptor-knockout animals are used because receptor compensation can affect disease progression.

## MATERIALS AND METHODS

### Animals

Isogenic male BALB/c, C57 black/6 and C57 black/6 B2RBK-knockout mice, aged 8–12 weeks (23–28 g), were obtained from the Animal Care Facility at the Federal University of São Paulo (UNIFESP). All animals were housed in individual standard cages and had free access to water and food. All procedures had been previously reviewed and approved by the internal ethical committee of the institution, adhering to the National Institutes of Health Guide for the Care and Use of Laboratory Animals, or equivalent.

### Induction of FSGS

FSGS was induced by a single tail-vein injection of Adriamycin (ADM; doxorubicin hydrochloride; Pfizer, New York, NY) (Wang et al., 2000b; Zheng et al., 2006). In the balb/c mouse background, a 10 mg/kg dose of ADM was injected to induce the disease (Pereira et al., 2011; Pereira et al., 2012). In the C57black/6 strain, a higher dose of 25 mg/kg ADM was used (Jeansson et al., 2009). An equal volume of saline was given to the control groups.

### Modulation of B2RBK in FSGS

B2RBK was modulated using different protocols. First, for the balb/c mice, the animals were treated with an intravenous injection of the B2RBK antagonist HOE-140 (Sigma, St Louis, MO) (30 μg per animal) (Zuccollo et al., 1996a; Zuccollo et al., 1996b) on days 1–3 after ADM injection. The animals were killed on days 4 and 21. In the second protocol (delayed treatment), the animals received HOE-140 on days 4–6 and were killed on days 7 and 21.

### Modulation of B1RBK

For the C57 black/6 strain, FSGS was induced by a single dose of ADM in wild-type and B2RBK-knockout mice. On days 1 and 6 after ADM injection, the animals were treated intraperitoneally with 10 mg/kg (Pereira et al., 2011) of the specific B1RBK receptor antagonist des-arg_9_-leu_8_-BK (DALBK) (Sigma, St Louis, MO).

### Renal function analyses

On days 1, 4, 7 and 21 after ADM injection, urinary and blood samples were collected. The urinary protein:creatinine ratio and albuminuria were used to estimate renal and podocyte function. At the time of killing, blood and urine were collected. All samples were analyzed using Labtest Diagnosis (Belo Horizonte, State of Minas Gerais, Brazil) and Sensiprot for protein measurements. To estimate the urinary albumin concentration, 10 μl of urine (adjusted to 1 mg/ml), corrected for urinary creatinine level, was separated by 10% SDS-PAGE and stained with Coomassie Blue.

The density of the bands was analyzed using the GeneSnap and Gene Tools software (Syngene, Cambridge, UK).

### Serum cytokine measurement

A Bio-Plex mouse cytokine assay kit (Bio-Rad) was used to test samples for the presence of kidney tissue cytokines. The assay was read on a Bio-Plex suspension array system, and the data were analyzed using Bio-Plex Manager software version 4.0. Standard curves ranged from 32,000 to 1.95 pg/ml.

### Determination of active TGF-β protein levels

Active TGF-β protein was measured using a TGF-β E_max_ immunoassay system (Promega), according to the manufacturer’s instructions. The results are presented as TGF-β pg/mg of total protein, measured using the Bradford assay (Bio-Rad, Hercules, CA).

### Gene expression

Kidney samples were frozen in liquid nitrogen. Total RNA was isolated using TRIzol Reagent (Invitrogen, Carlsbad, CA).

First-strand cDNAs were synthesized using Moloney murine leukemia virus reverse transcriptase (Promega, Madison, WI).

Real-time PCR was performed using TaqMan primers and probes for NPHS-1 (TaqMan probe Mm004497831_g1), vimentin (TaqMan probe Mm 00801666-g1), TNF-α (TaqMan probe Mm0136932), PAI-1 (TaqMan probe Mm 009312), CTGF (TaqMan probe Mm01192932), WT-1 (TaqMan probe Mm 01337053_m1), IL-1β (TaqMan probe Mm00434228), TGF-β (TaqMan probe Mm01178820) and COL-1 (TaqMan probe Mm00801666) (Applied Biosystems, Foster City, CA). For the analyses of B1RBK, B2RBK, NPHS-2, α-ACTININ-4 and MCP-1, real-time PCR was performed using a SYBR green assay (Applied Biosystems; Table 1).

**Table 1.**
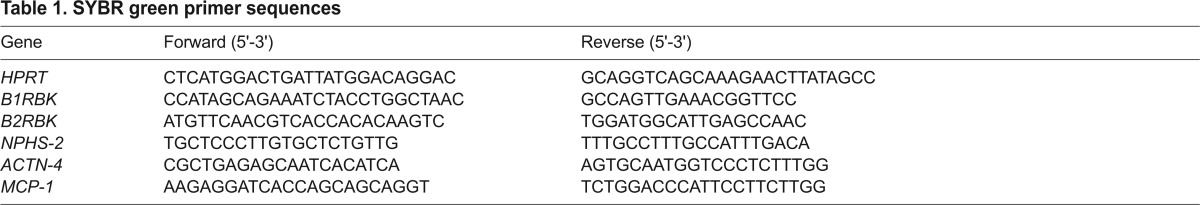
SYBR green primer sequences

The cycling conditions for both TaqMan and SYBR green primers were as follows: 10 minutes at 95°C, followed by 45 cycles of 30 seconds at 95°C, 30 seconds at 60°C and 30 seconds at 72°C. The relative quantification of mRNA levels was performed as described in detail in User Bulletin 2 (PerkinElmer, Applied Biosystems, Branchburg, NJ, 1997). Briefly, the target gene amount was normalized to the endogenous reference [hypoxanthine phosphoribosyltransferase 1 (HPRT); SYBR green] and then related to a calibrator (sample with the lowest expression, namely the controls) using the formula 2^−DDCt^. Hence, all data that are expressed as an n-fold difference are related to the expression of matched controls. Analyses were performed with the Sequence Detection Software 1.9 (Applied Biosystems, Foster City, CA).

### Western blotting

Briefly, 50 μg of total protein from renal tissue was collected and then diluted in sample buffer (Bio-Rad) containing 20 mg/ml of 2-β-mercaptoethanol (Sigma, St Louis, MO). The antibodies used were against β-actin (1:1000; Sigma, St Louis, MO) and NPHS-1 (1 ug/mL; Abcam, Cambridge, UK). Western blotting was performed according to the Abcam manufacturer’s protocols found at www.abcam.com/ps/pdf/protocols/WB-beginner.pdf.

### Renal histology analysis

Kidney samples were fixed in 10% neutral formalin. Paraffin sections (3 mm in thickness) were cut and stained with hematoxylin and eosin. The sections were analyzed by using a trinocular optical microscope (Olympus Corporation, Tokyo, Japan). Photographs were taken by using a digital camera that was coupled to the microscope, and the images were captured by using with the Pinnacle Studio Plus software (Pinnacle Systems, Buckinghamshire, UK). Glomerulosclerosis was evaluated as described previously (Mu et al., 2005). The extent of glomerulosclerosis and glomerular collapse was evaluated in each kidney by consecutive examination under a light microscope. Tubulointerstitial injury was defined as tubular dilation and/or atrophy or as interstitial fibrosis (Zeisberg and Kalluri., 2004).

Tubular injuries were examined in at least 20 areas using the following scoring system: 0, changes in <10% of the cortex; 1+, changes in up to 25% of the cortex; 2+, changes in up to 50% of the cortex; and 3+, changes in 50% of the cortex sections.

### Transmission electron microscopy analysis

1-mm^3^ of renal tissue was collected and fixed by incubation for 2 hours in 1.5% glutaraldehyde and 1% paraformaldehyde in cacodylate buffer (0.1 M, pH 7.3). The tissue was post-fixed in 1% of osmium tetroxide in cacodylate buffer (0.1 M, pH 7.3). After a series of ethanol dehydrations, the tissue was resin-embedded. Ultrafine sections were sliced and colored with uranyl acetate and lead citrate, and then exanimated and micro-photographed.

### Statistical analysis

All data are presented as the mean±s.e.m. The differences among three or more groups were compared using analysis of variance (ANOVA) with a Tukey post-test. When two groups were compared, unpaired Student’s *t*-tests were used. Significance was established as *P*<0.05. All statistical analyses were performed using GraphPad PRISM (GraphPad, La Jolla, CA).

## Supplementary Material

Supplementary Material
