# Supplementary material for: Balance between the two kinin receptors in the progression of experimental focal and segmental glomerulosclerosis in mice
Source: Dis Model Mech. 2014 Apr 17;7(6):701–10. doi: 10.1242/dmm.014548 (PMC4036477; doi:10.1242/dmm.014548)
Supplement: Supplementary Material [file supp_7_6_701__index.html]

Balance between the two kinin receptors in the progression of experimental focal and segmental glomerulosclerosis in mice — Supplementary Material 

# Balance between the two kinin receptors in the progression of experimental focal and segmental glomerulosclerosis in mice

## DMM014548 Supplementary Material

**Files in this Data Supplement:**

- **Supplementary Material**
