# Supplementary material for: Balance between the two kinin receptors in the progression of experimental focal and segmental glomerulosclerosis in mice
Source: Dis Model Mech. 2014 Apr 17;7(6):701–10. doi: 10.1242/dmm.014548 (PMC4036477; doi:10.1242/dmm.014548)
Supplement: Supplementary Material [file supp_7.6.701_DMM014548.pdf]

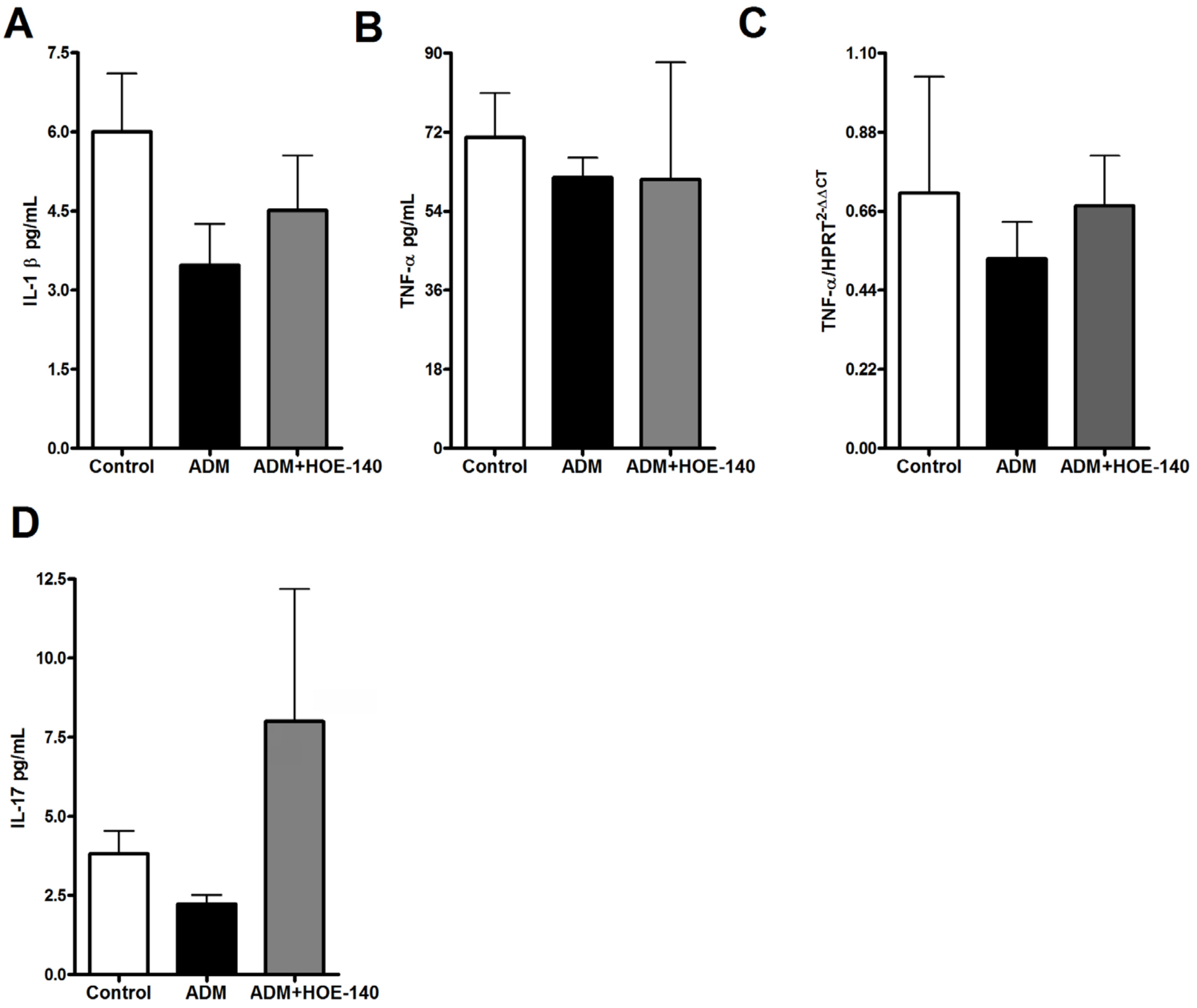

Supplemental Fig 1. Early HOE-140 treatment did not alter the expression of some podocyte and inflammatory cytokines. The mice were treated with HOE-140 at days +1, +2 and +3 after ADM injection, and were sacrificed at day 4. HOE-140 treatment did not alter the mRNA expression of nephrin (A) or alpha-actinin 4 (B). HOE-140 treatment also did not alter the renal protein expression of the pro-inflammatory cytokine IL-1β(C), the renal protein (D) and mRNA (E) expression of TNF-βor the renal protein expression of IL-17 (F). Abbreviations: ADM (balb/c mice treated with Adriamycin); HOE-140 (B2 receptor antagonist); NPHS-1 (nephrosis 1, idiopathic, steroid-resistant); ACTN-4 (actinin, alpha 4); IL-1β(Interleukin-1β; TNF-β(Tumor necrosis alpha); IL-1β(interleukin1-β; and IL-17 (interleukin 17). * indicates $P<0.05$ vs. control; and # indicates $P<0.05$ vs. ADM. Five animals were used per study group.

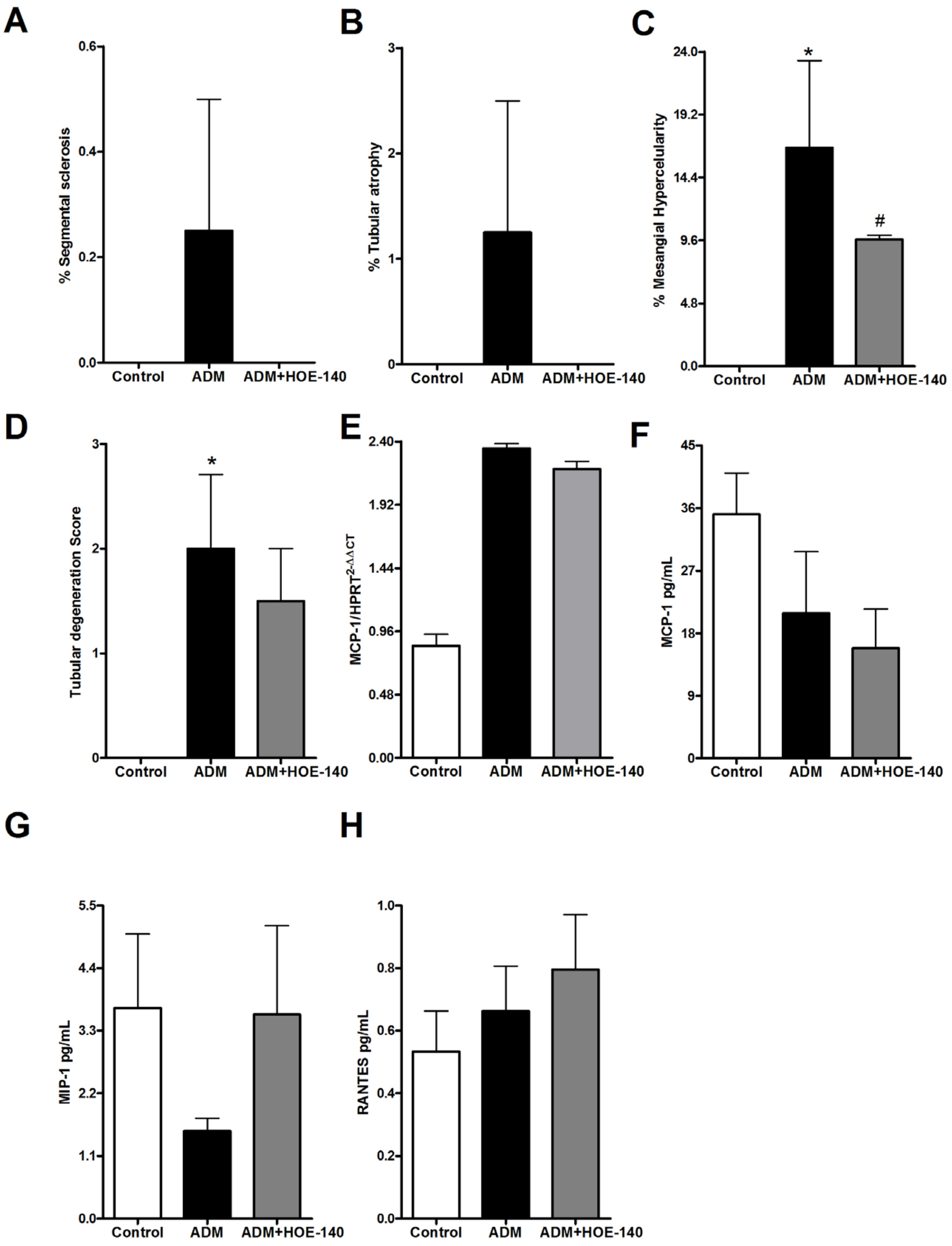

Supplemental Fig 2. Early HOE-140 treatment protects animals from mesangial hypercellularity induced by Adriamycin injection. The mice were treated with HOE-140 at days +1, +2 and +3 after ADM injection, and the animals were sacrificed at day 4. HOE-140 treatment did not affect the renal sclerosis index (A) or tubular atrophy (B) induced by ADM injection. However, HOE-140 treatment reduced the mesangial hypercellularity index (C) but did not affect the tubular degeneration score (D). The levels of macrophage-related chemokines were not different between the groups (F-H). Abbreviations: ADM (balb/c mice treated with Adriamycin); HOE-140 (B2 receptor antagonist); MCP-1 (macrophage chemoattractant protein-1); MIP-1 (macrophage inflammatory protein 1-alpha); and RANTES (Regulated on Activation, Normal T cell Expressed and Secreted). * indicates P<0.05 vs. control; and # indicates P<0.05 vs. ADM. Five animals were used per study group.

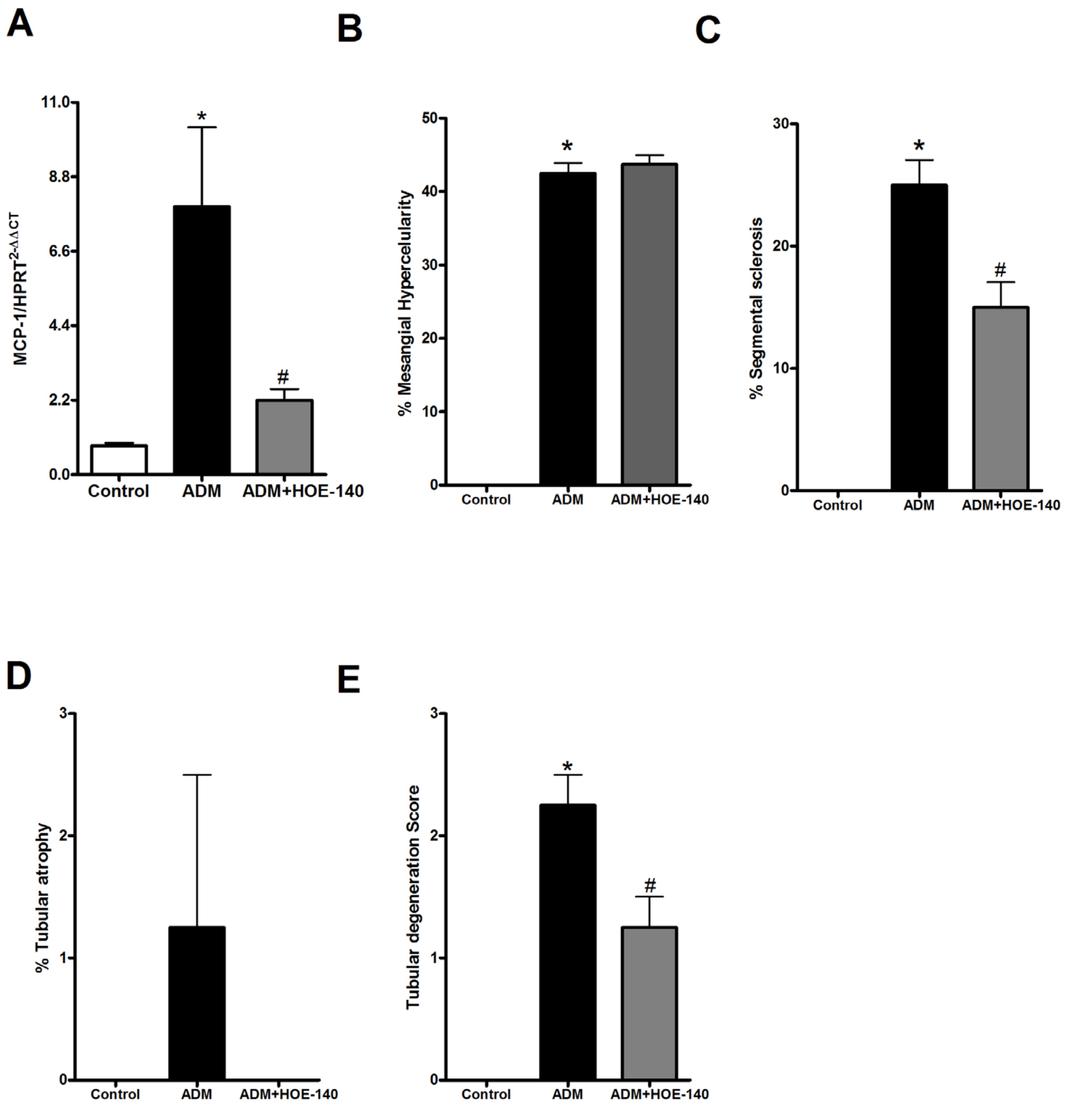

Supplemental Fig 3. Delayed HOE-140 treatment protects animals from segmental sclerosis and MCP-1 upregulation induced by Adriamycin injection. Then, the mice were treated with HOE-140 at days +4, +5 and +6 after ADM injection, and were sacrificed at day 7. HOE-140 treatment diminished the levels of MCP-1 mRNA (A). HOE-140 did not affect the mesangial hypercellularity index (B). However, HOE-140 treatment reduced the levels of segmental sclerosis (C), did not significantly affect the levels of tubular atrophy (D) and downregulated the tubular degeneration scores (E). Abbreviations: ADM (balb/c mice treated with Adriamycin); HOE-140 (B2 receptor antagonist); MCP-1 (macrophage chemoattractant protein-1); MIP-1 (macrophage inflammatory protein 1-alpha); and RANTES (Regulated on Activation, Normal T cell Expressed and Secreted). * indicates $P<0.05$ vs. control; and # indicates $P<0.05$ vs. ADM. Five animals were used per study group.

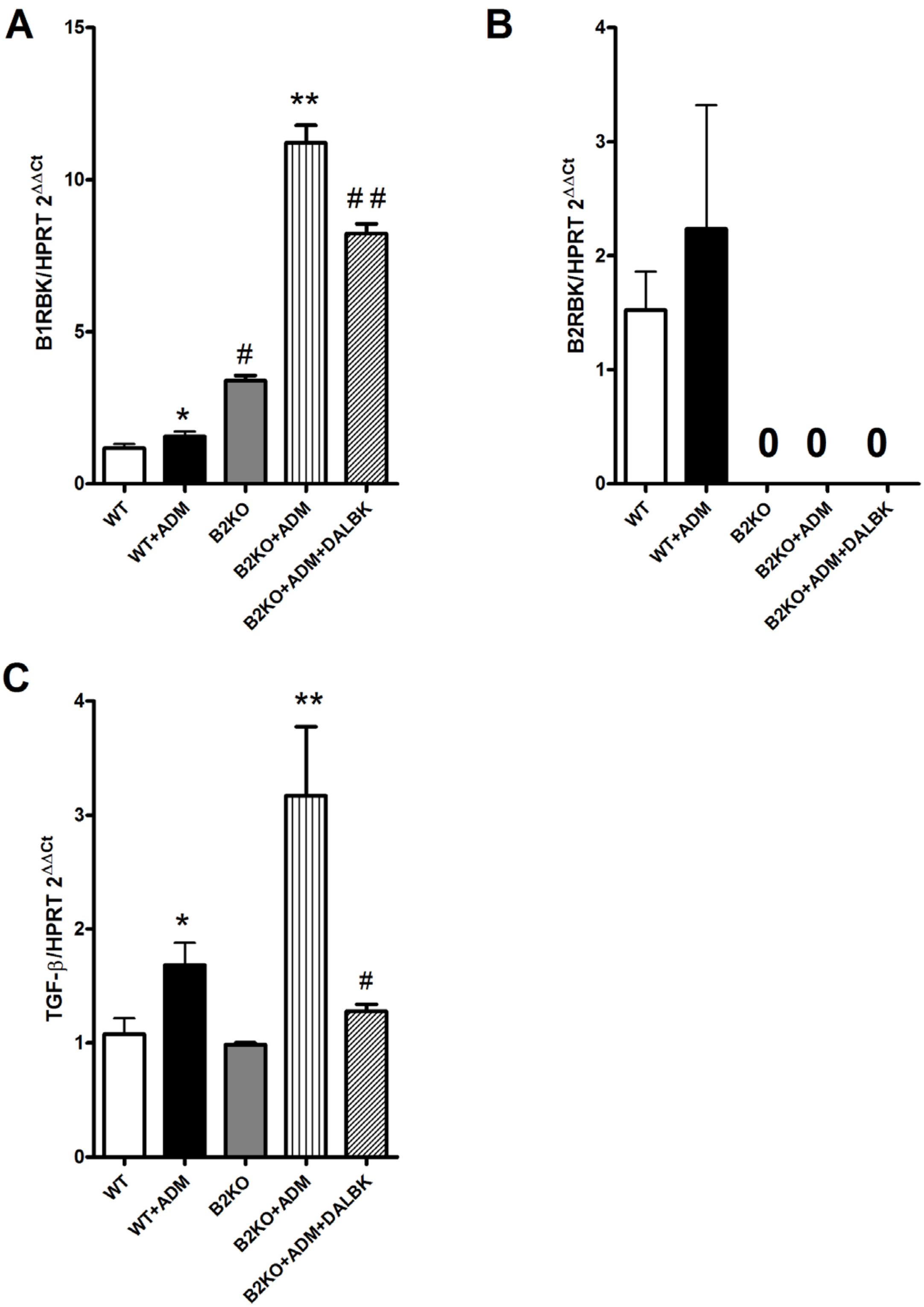

Supplemental Fig 4. The lack of the B2RBK receptor exacerbates the upregulation of B1RBK and TGF-β B2RBK KO mice were then treated daily with the B1RBK antagonist DALBK (des-arg9-leu8-BK). At the end of the protocol, the mice were sacrificed at day 7. The lack of B2RBK exacerbates ADM-induced B1RBK expression in WT mice that was exacerbated in B2RBK KO animals, and it was expressed less with DALBK treatment (A). The levels of B2RBK were not different between the groups, and B2RBK was not detected in B2RBK KO mice (B). The mRNA expression of TGF-βwas upregulated after ADM injection in WT mice and even highly expressed in B2RBK KO animals challenged with ADM. Furthermore, this expression was reduced after DALBK treatment (C). Abbreviations: ADM (C57black background mice treated with Adriamycin); B2RBK (bradykinin receptor 2) B1RBK (bradykinin receptor 1); TGF-β (transforming growth factor, beta 1); HPRT (hypoxanthine guanine phosphoribosyl transferase); and βActin (actin, beta). * indicates $P<0.05$ vs. control; and # indicates $P<0.05$ vs. ADM. Five animals were used per study group.
